# The Wnt pathway induces a naïve-like subpopulation in primed stem cells, while NME7_AB_ leads to a homogeneous naïve-like population

**DOI:** 10.1371/journal.pone.0325997

**Published:** 2025-06-25

**Authors:** Kevin R. Yi, Jac-Leen S.S. Nash, Mark G. Carter, Danica M. Walkley, Jiwon Jang, Benoit J. Smagghe, Andrew K. Stewart, Cynthia C. Bamdad

**Affiliations:** 1 Minerva Biotechnologies, Waltham, Massachusetts, United States of America; 2 Department of Life Sciences, Pohang University of Science and Technology (POSTECH), Pohang, Republic of Korea; University of Colorado Boulder, UNITED STATES OF AMERICA

## Abstract

The literature is replete with conflicting reports as to whether the Wnt/β-catenin pathway induces human stem cell differentiation or pluripotency. Recently, scientists showed that human stem cells expressing low levels of active β-catenin preferentially differentiate down a neuroectoderm lineage, whereas cells expressing high levels favor mesendoderm. However, these results appear to contradict two other studies, where researchers improved differentiation to both neuroectoderm and mesoderm by increasing levels of active β-catenin at the start of differentiation. Here, we show that stem cells cultured with naïve growth factor, NME7_AB_, express the highest levels of active β-catenin, yet readily differentiate into neuroectoderm and mesendoderm, without lineage preference. This raised the interesting question of whether activation of the Wnt/β-catenin pathway could itself play a role in maintaining or inducing a naïve-like state. The β-catenin agonist WNT3A was added to stem cells in the absence of any other growth factors, which induced the concurrent emergence of two segregated populations: an OCT4^+^, XaXa naïve-like population and an OCT4^-^ population. This finding could explain the apparently inconsistent reports as to whether β-catenin induces pluripotency or differentiation, while raising additional questions. Notably, does the naïve-like sub-population, devoid of cell fate decisions, contribute to an increased differentiation potential? Conversely, are the OCT4^-^ cells predisposed to differentiate more efficiently? To address these questions, we compared the differentiation of primed state stem cells, with or without pre-treatment with WNT3A, to that of naïve state stem cells. WNT3A pre-treatment improved the differentiation potential of primed stem cells, while having no effect in naïve stem cells. Furthermore, differentiation of the homogeneous population of naïve cells was superior to the primed state cells, even after WNT3A pre-treatment. This result is consistent with the idea that the improved differentiation is due to the sub-population of the WNT3A induced naïve-like cells.

## Introduction

The role of Wnt/β-catenin signaling in stem cell pluripotency and differentiation is incompletely understood. Several studies report that Wnt signaling promotes pluripotency while others report that it induces differentiation [1–13]. Jang et al. [14] recently reported that the media in which stem cells are grown influences expression levels of active β-catenin, which affects the length of the G1 phase of the cell cycle, which in turn affects differentiation preferences. Cells that express low levels of active β-catenin have a relatively long G1 phase and preferentially differentiate along a neuroectoderm lineage, while cells that express high levels have a shorter G1 phase and preferentially differentiate to mesendoderm lineage. Here, we extend the investigation of the role of Wnt/β-catenin signaling in stem cell pluripotency and differentiation, using multiple types of pluripotent stem cells, including naïve state stem cells [15–20]. Naïve stem cell populations were generated and maintained by culturing in a minimal, serum-free media containing NME7_AB_ as the only growth factor. NME7_AB_ is secreted by every cell of the inner cell mass of an early human blastocyst. NME7_AB_ binds to and dimerizes the MUC1* growth factor receptor to promote naïve pluripotency [16,17,21].

## Materials and methods

### Stem cell lines

iPS_NME7-6E_ and iPS_NME7-N7B_ were generated as previously described [16] from BJ fibroblast cells (ATCC CAT# CRL2522), using the Yamanaka core pluripotency factors CytoTune system in NME7_AB_ naïve media.

iPS_NME7-9X_ were generated from frozen peripheral blood mononuclear cells (PBMCs) (AllCells NPB-MNC CAT# PB003F) using a protocol based on Thermo Fisher’s CytoTune 2.0 system. Key differences include the use of NME7_AB_ in the media starting from the thaw of the PBMCs, the use of NME7_AB_ naïve media instead of the CytoTune iPSC media, and colonies were picked onto vitronectin and subsequently passaged on anti-MUC1*mouse monoclonal antibody MN-C3 coated plates instead of mouse embryonic fibroblasts (MEFs).

iPS_FGF-A6_ cells were generated from BJ fibroblast cells using the Yamanaka core pluripotency factors CytoTune system.

hES_HES3_ cells were obtained from ESI Bio.

### Stem cell culture

Naïve stem cells were cultured in AlphaStem (Minerva Biotechnologies, Waltham, MA), which is a minimal media containing 4nM recombinant NME7_AB_ over a surface of anti-MUC1* mouse monoclonal antibody MN-C3. iPS_FGF-A6_ cells were cultured in either E8 on vitronectin or mTeSR1 on Matrigel. hES_HES3_ cells were grown on irradiated mouse embryonic ﬁbroblasts (MEFs) in 4–10 ng/mL FGF2 (R&D Systems, Minneapolis, MN) and in some cases Rho kinase inhibitor (Y-27632, SelleckChem) was added. Cells were maintained at 37^o^C in 5% CO_2_/5% O_2_.

### Testing the effect of WNT3A in the absence of other growth factors

Stem cells were cultured in their respective media for at least 12 passages. Cells were re-plated onto gelatin in AlphaSTEM minimal media to which was added 100ng/mL of WNT3A. Media was changed every 24 hrs. Cells were stained for OCT4 and tri-methylated Lysine 27 on Histone 3. Images were collected an Olympus IX81 using 4x, 10x, or 20x objectives. We note that cells grown in mTeSR1 and E8 could not be sustained on gelatin and were re-plated on GFR-Matrigel and Fibronectin respectively.

### Differentiation assays

Measurement of dopamine and its metabolites was performed at the Vanderbilt University Neurochemistry Core by HPLC. Alcian blue staining of chondrocytes was performed at Brigham and Women’s Hospital Pathology Core. All antibodies and assay kits are listed in the S1 Table.

## Results

### Does the Wnt/β-catenin pathway promote a naïve-like stem cell state?

**Fig 1:**
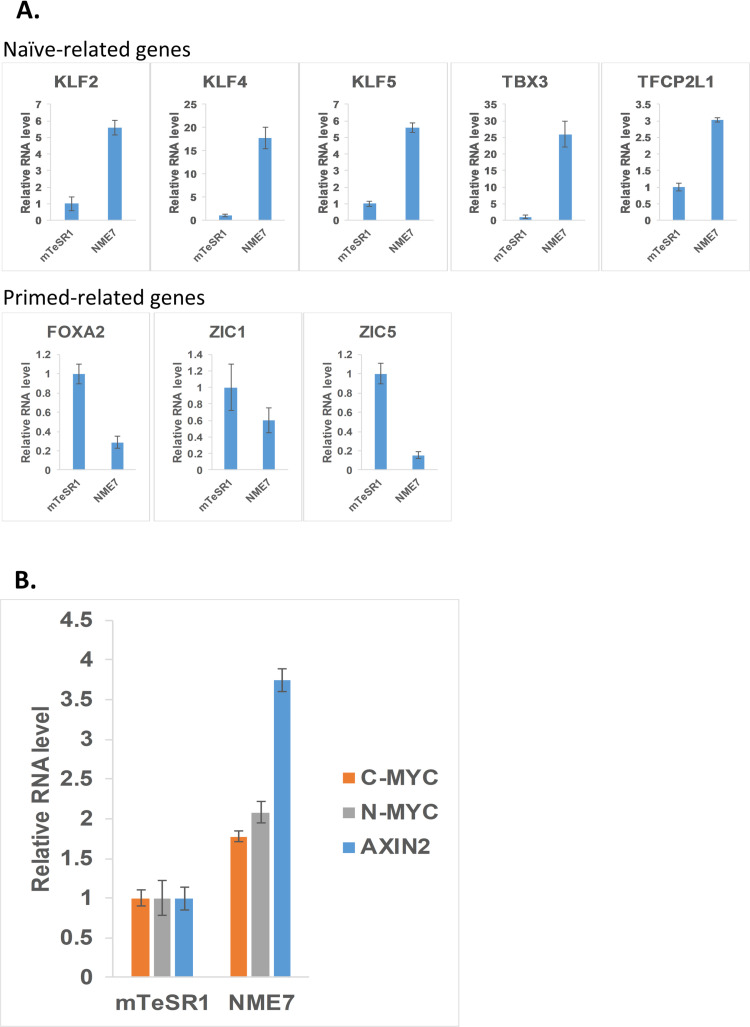
Characterization of hESCs after culture in either mTeSR1 media or NME7_AB_ naïve media. (A) qPCR analysis of naïve and primed markers in H9 cells grown either in mTeSR1 or in NME7_AB_ (n = 4). (B) qPCR analysis of WNT target genes in H9 cells grown either in mTeSR1 or in NME7_AB_ (n = 4).

**Fig 2:**
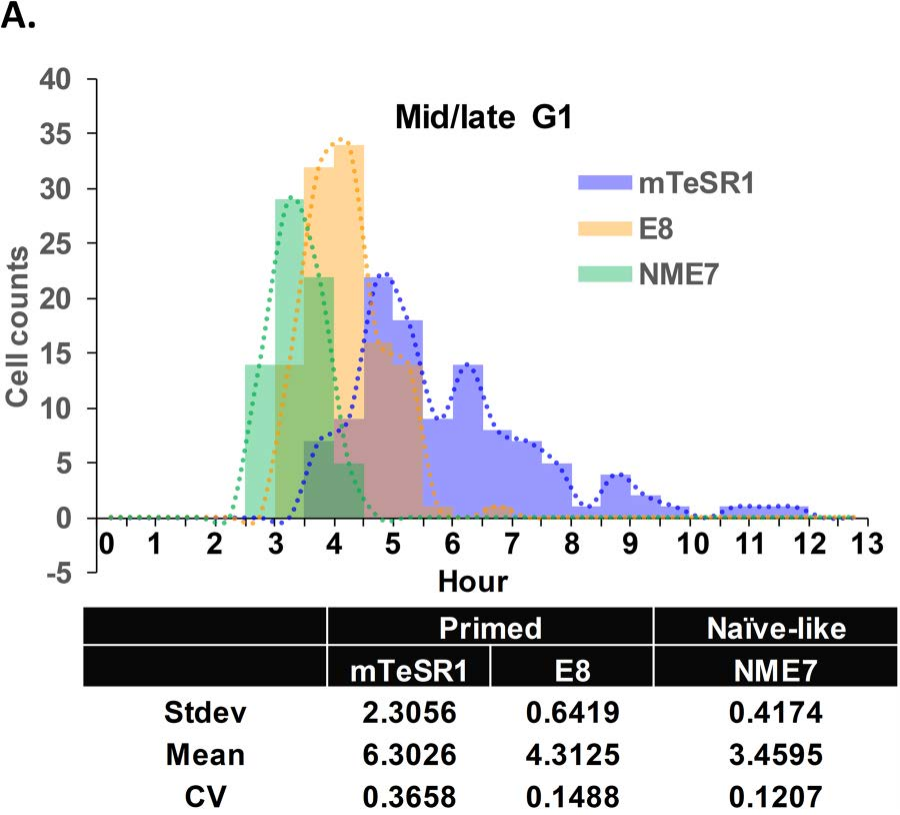
G1 length distribution in hESCs cultured in different media. **(A)** Histograms for mid/late G1 length of H9 cells grown in mTeSR1, E8, or NME7_AB_ (n = 114 for mTeSR1, n = 112 for E8, and n = 70 for NME7_AB_ from three independent experiments). KS test: p-value = 1.554e-15 from NME7_AB_ vs. E8 and p-value < 2.2e-16 for NME7_AB_ vs. mTeSR1.

It was previously reported that stem cells cultured in E8 media express high levels of β-catenin and cells cultured in mTeSR1 have low levels [14]. Here we show that the same stem cells cultured in NME7_AB_ naïve media express naïve-related genes and also express the highest levels of active β-catenin (Fig 1A and 1B) [14]. Stem cells grown in mTeSR1, which express low β-catenin, preferentially differentiated to neuroectoderm cells, while cells grown in E8, expressing higher β-catenin, do not. Conversely, E8 grown cells preferentially differentiated along mesendodermal lineages. NME7_AB_ grown naïve stem cells express the highest levels of active β-catenin and have the shortest G1 phase (Fig 2) [14].

**Fig 3:**
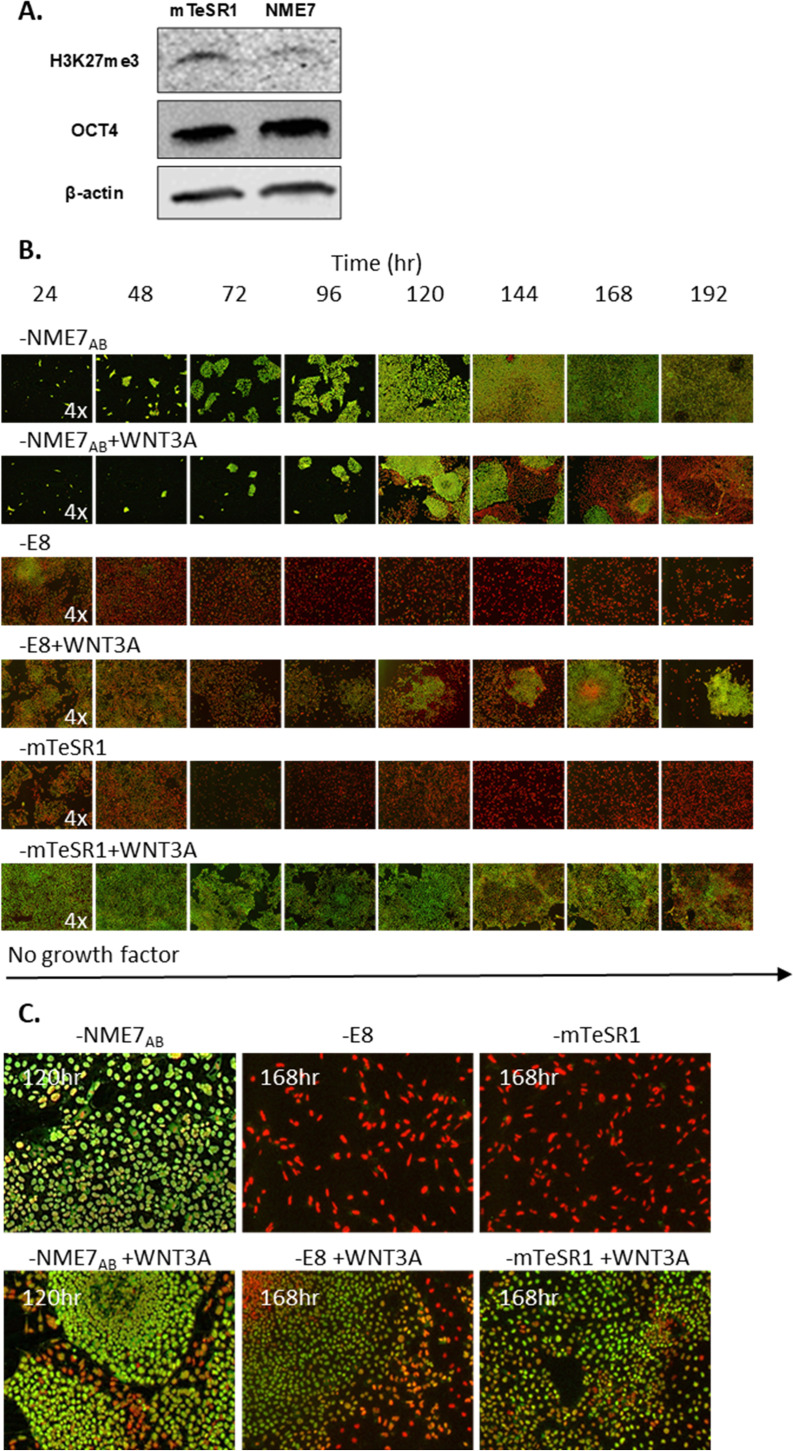
Tracking X chromosome activation status and pluripotency in response to stimulating **β-catenin signaling.** (A) The amount of tri-methylated Lysine 27 on Histone 3 (H3K27me), an indicator of inactivation of an X chromosome, was quantified by Western blot for pluripotent stem cells that had been previously cultured in either mTeSR1 or NME7_AB_ media. **(B)** The expression patterns of H3K27me and OCT4 were fluorescently imaged over an 8-day time course. iPSCs that had previously been cultured in either NME7_AB_ naïve media, E8 or mTeSR1 were switched to a growth factor-free media in the presence or absence of β-catenin agonist WNT3A. Cells were double stained for OCT4 (green) and tri‐methylated Lysine 27 on Histone 3 (H3K27me) (red). H3K27me stain is absent or cloud‐like if both X chromosomes are active (XaXa), but appears as a condensed dot if one X has been silenced (XaXi). Between 120 and 192 hours after respective growth factors were removed and WNT3A was added, two populations emerge: a first OCT4 positive population that lacks the punctate H3K27me staining, which is characteristic of a naïve state, and a second OCT4 negative population that has the dark or punctate H3K27me staining, indicative of a differentiating or primed state. **(C)** Magnified images underscore the effect of activating the β-catenin/Wnt pathway in the absence of other growth factors.

The finding that naïve stem cells express the highest levels of β-catenin and do not spontaneously differentiate suggests a role for the Wnt/β-catenin pathway in maintaining or inducing a naïve stem cell state. To test this hypothesis, we used female stem cells so that the X-activation status, an indicator of naïve state, could be tracked in response to the addition of the β-catenin agonist, WNT3A. Recall that female stem cells have two active X chromosomes (XaXa) in the naïve state, but in the later primed state, one of the X chromosomes is inactivated (XaXi) [15,18–20]. When primed state stem cells are stained with an antibody that binds to tri-methylated Lysine 27 on Histone 3 (H3K27me), fluorescence imaging shows a punctate red dot in the nucleus, whereas in naïve state stem cells H3K27me staining is absent or appears as a weak cloud-like stain [22,23]. Western blot analysis confirms decreased expression of the tri-methylated Lysine in NME7_AB_ grown cells compared to mTeSR1 cells (Fig 3A) [16]. Double staining for OCT4 positivity and X-activation status provides a convenient method for tracking stem cells as they enter or exit naïve state and/or pluripotency.

Female stem cells were grown for 12 passages in either NME7_AB_ naïve media (iPSC_NME7-9X_), E8 media (iPSC_E8-9G_) or mTeSR1 (iPSC_mTeSR1-9G_) such that they start off in either a naïve or a primed state, respectively [16]. To see if a β-catenin agonist could induce a naïve-like state, the pluripotent stem cells were switched to a growth factor-free media to which was added WNT3A. Over an 8-day time course, OCT4 positivity was tracked as an indicator of pluripotency and X chromosome activation status was followed by H3K27me staining, as an indicator of a naïve state (Figs 3B and 3C). Surprisingly, when NME7_AB_ was withdrawn from naïve state cells, the addition of WNT3A simultaneously induced two separate and distinct populations: islands of OCT4 positive cells that lacked the H3K27me staining, indicative of a pluripotent and naïve state, in a sea of OCT4 negative cells with the punctate H3K27me staining that is indicative of a differentiating state. Stem cells that had been previously cultured in E8 media rapidly lost OCT4 positivity when switched to a growth factor-free media. However, in the presence of WNT3A, a sub-population of OCT4 positive, perhaps XaXa naïve-like cells emerged. The WNT3A effect appears to be delayed on stem cells previously grown in mTeSR1. Whereas cells previously grown in NME7_AB_ or E8 media were able to grow on gelatin, cells that had been grown in mTeSR1 could not and needed to be plated onto Matrigel. The numerous growth factors present in Matrigel could have confounded the effect of WNT3A.

If activating the Wnt/β-catenin signaling pathway reverts a sub-population of cells to a naïve state, and if naïve state stem cells differentiate better than primed state stem cells, then the prediction is that the addition of a β-catenin agonist would improve the differentiation potential of primed state cells, but have no effect on stem cells that are already in a naïve state. To test that hypothesis, we grew human iPSCs in NME7_AB_, E8 or mTeSR1, then treated the cells with either WNT3A, a β-catenin agonist, or JW67, a β-catenin inhibitor, for 48 hours before initiating differentiation to: 1) retinal progenitor cells; 2) mesenchymal stem cells; and 3) hepatocytes. When differentiating the stem cells to dopaminergic neurons, neither β-catenin agonist or inhibitor was added because the protocol begins with the addition of CHIR99021, which is an indirect agonist of β-catenin.

### The effect of stimulating versus inhibiting the β-catenin pathway on neuroectoderm differentiation

**Fig 4:**
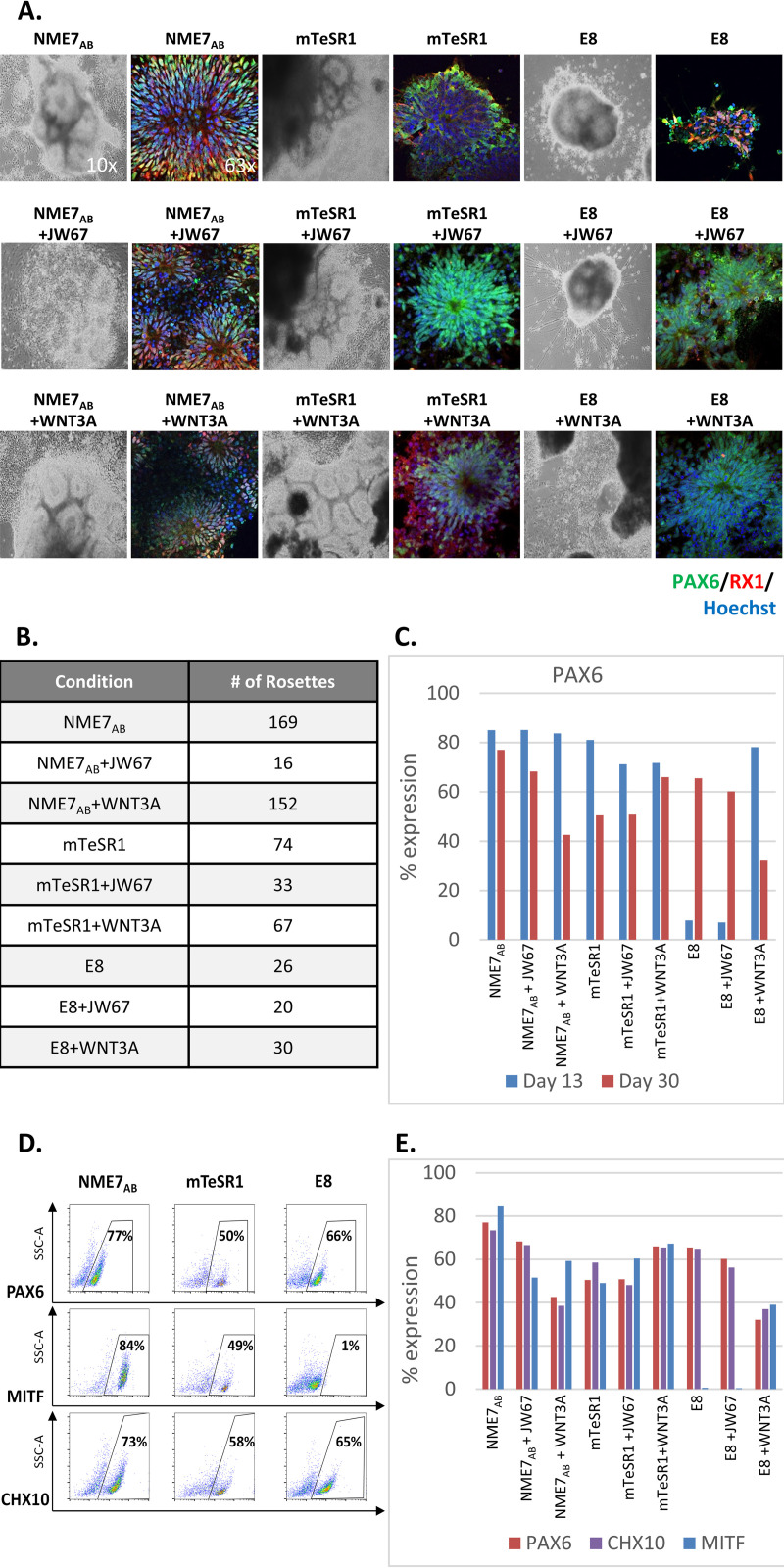
Stimulating versus inhibiting the **β-catenin pathway in retinal progenitor cell differentiation.** Retinal progenitor cells (RPCs) were differentiated from human iPSCs that had been cultured in either NME7_AB_ naïve media, mTeSR1 or E8, with or without 48 hours of pre-culture in the β-catenin inhibitor JW67 or the β-catenin agonist WNT3A. **(A)** Bright field (10x) and immunofluorescent images (63x) of neural rosettes at Day 30 were stained for the presence of PAX6 (green), a marker of neuroectoderm cell fate and RX1 (red), a marker of retinal precursors, with nuclei stained blue with Hoescht-33342. **(B)** Table lists the average number of neural rosettes per condition for 1 well of a 6-well plate. **(C)** A graph of flow cytometry measurement shows the contrast between the percent of the cell populations that expressed PAX6 at Day 13 versus Day 30, for each test condition. **(D)** Day 30 flow cytometry scatter plots highlight the differences in expression of PAX6 and two critical markers of retinal progenitor cell differentiation, MITF and CHX10, when iPSCs previously cultured in either NME7_AB_, mTeSR1 or E8 media are differentiated to RPCs. **(E)** The bar graph of flow cytometry measurement of PAX6, CHX10 and MITF at Day 30 shows the effect of β-catenin antagonist JW67 versus the effect of the agonist WNT3A, particularly on the expression of MITF.

Induced pluripotent stem cells (iPSCs) that had been cultured in either NME7_AB_ naïve media, E8 or mTeSR1, plus a β-catenin agonist or inhibitor were differentiated into retinal progenitor cells (RPCs) according to the protocol from Barnea-Cramer et al. [24] (S1A Fig). At the neural rosette stage, significant differences were observed in the ability of the cells to express PAX6, a marker of neuroectoderm cell fate and RX1, a marker of retinal precursors. Additionally, there were pronounced differences in their ability to form the characteristic rosette morphology, consisting of flat cells organized into well-ordered radial structures resembling flowers (Fig 4A; S1B Fig). Consistent with the findings of Jang et al., stem cells that had been grown in mTeSR1, which express low levels of β-catenin, readily differentiated into abundant clusters of properly structured neural rosettes. In contrast, stem cells previously grown in E8 media, which express high levels of β-catenin, did not. The addition of β-catenin antagonist JW67 for 48 hours before initiating differentiation improved the outcome with a few properly structured rosettes being formed. However, adding WNT3A to E8 media before initiating differentiation unexpectedly improved the outcome even more, with the formation of numerous clusters of neural rosettes. Stem cells that had been cultured in NME7_AB_ naïve media, which express the highest levels of β-catenin, readily differentiated into the highest number of neural rosettes (Fig 4B), having some of the largest diameters and displaying the characteristic rosette morphology. The addition of β-catenin agonist or inhibitor did not have a significant effect on the differentiation of stem cells previously grown in NME7_AB_ naïve media. Flow cytometry revealed that, without pre-culture in WNT3A, stem cells that had been cultured in E8 media had delayed expression of PAX6, an early neuroectoderm marker (Fig 4C; S1C Fig). In the development of the eye, transcription factors CHX10 and MITF have mutually exclusive expression patterns, with CHX10 marking optic vesicle cells destined to form the retina, while MITF marks cells for retinal pigmented epithelium (RPE) [25–27]. MITF is highly overexpressed in RPE cells and is considered a master regulator of RPE differentiation [28,29]. Without the addition of WNT3A, stem cells previously grown in E8 do not express MITF (Fig 4D and 4E). Compared to when JW67 was added to the basal media, WNT3A addition led to higher levels of MITF (Fig 4E).

**Fig 5:**
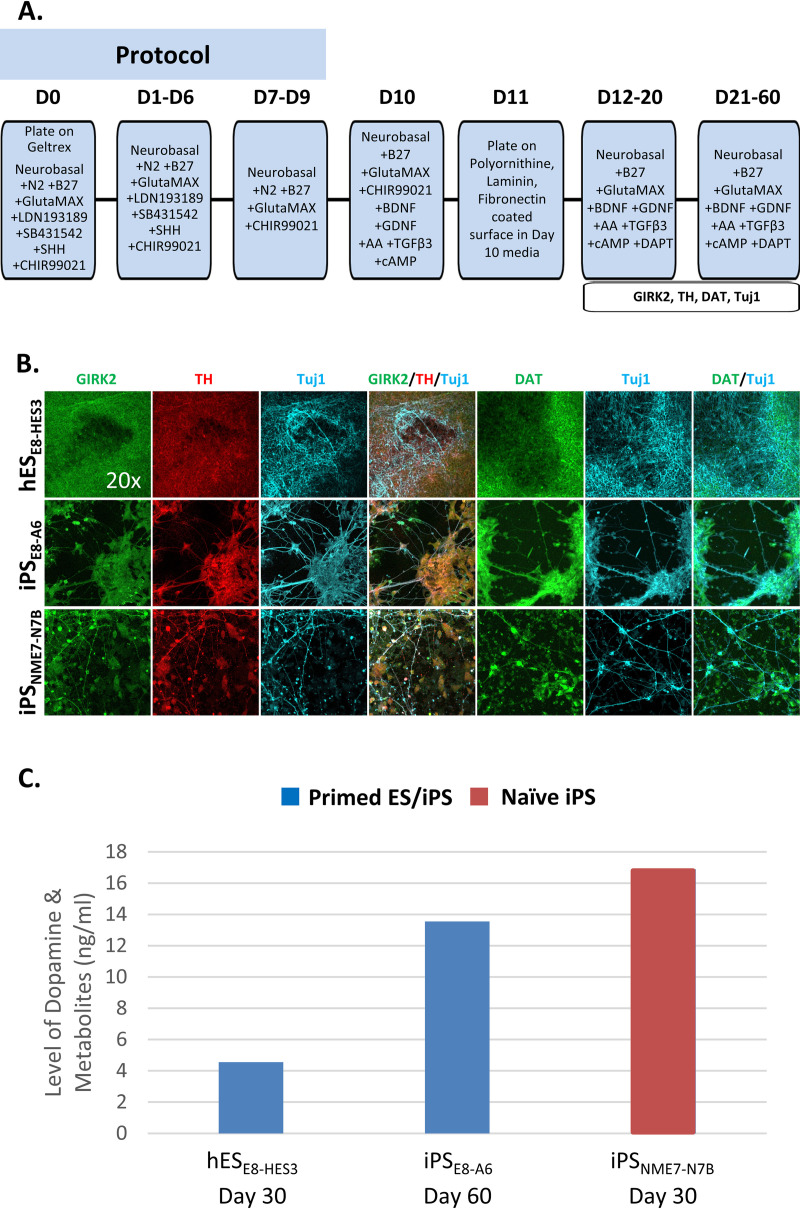
Dopaminergic neuron differentiation with different starting cell types or media. Dopaminergic neurons were differentiated from human ESCs and iPSCs that had been grown in E8 medium (hES_E8-HES3_; iPS_E8-A6_) and also from iPSCs that had been grown in NME7_AB_ naïve media (iPS_NME7-N7B_). **(A)** Cartoon of protocol that was followed. **(B)** Fluorescent images of resultant cells at Day 24 show differences in cell morphology and expression of key molecular markers of dopaminergic neurons. (C) The spontaneous release of dopamine and its metabolites (3,4-dihydroxyphenylacetic acid, 3-methoxytyramine, and homovanillic acid) into the media was measured by HPLC. The graph shows the highest level that was measured for that cell type, based on 800,000 cells plated on Day 11 of differentiation, and the day post-differentiation that level was reached. For comparison, on Day 30 iPS_NME7-N7B_ cells secreted 16.90 ng/mL dopamine, while on Day 30 iPS_E8-A6_ cells only secreted 1.77 ng/mL.

We next generated dopaminergic neurons from human iPSCs that had been cultured in either NME7_AB_ naïve media or in E8 media. A protocol based on a publication by Kim et al. [30] was used (Fig 5A). Interestingly, this protocol starts by adding CHIR99021, a GSK3β inhibitor, which is an inhibitor of β-catenin. Because CHIR99021 is an indirect agonist of the Wnt/β-catenin pathway, neither WNT3A nor JW67 was added before initiating differentiation.

The resulting cells were analyzed for the presence of key molecular markers of dopaminergic neurons, including GIRK2 (G-protein-regulated inward-rectifier potassium channel 2), which is primarily expressed in A9 dopaminergic neurons and TH (tyrosine hydroxylase), often considered a gold standard in the identification of dopaminergic neurons because it is an enzyme that catalyzes the conversion of L-tyrosine to L-3,4-dihydroxyphenylalanine, which is the rate limiting step in dopamine synthesis [31]. The expression of DAT (dopamine active transporter) is equally important as it is the transmembrane protein that pumps dopamine from the synapse back into the cytosol. Tuj1 (neuron-specific class Ill B-tubulin) is a pan-neural marker. Since some clinical trials anticipate implanting dopaminergic neuron progenitors early in the differentiation process, we assayed for the presence of these key molecular markers at Day 24 of differentiation (Fig 5B). All three types of starting cells differentiated into cells that were positive for these molecular markers. However, there were significant differences in cell morphology and in their patterns of expression. The cells derived from embryonic HES3 cells previously grown in E8 stain positive for the specific markers of dopaminergic neurons, GIRK2, TH and DAT. However, those stains are not associated with cells having neural morphology. iPSCs that had been cultured in E8 stain positive for the markers that are specific to dopaminergic neurons and form neural projections, but they emanate from clumps of cells, of which few have neural morphology. The cells generated from iPSCs that had been grown in NME7_AB_ naïve media stain positive for GIRK2, TH, and DAT and importantly have more complex, interconnected networks of neural projections emanating from cells with characteristic elongated, triangular shape of neurons.

Perhaps the most important measure of dopaminergic neuron function is their ability to make and secrete dopamine. The amount of dopamine that was spontaneously secreted, from dopaminergic neurons that had been differentiated from iPSCs that were cultured in either E8 or NME7_AB_ naïve media, was measured by HPLC at different timepoints. The highest dopamine measurement for each cell type is shown in the graph of Fig 5C. Dopaminergic neurons derived from the iPSCs grown in NME7_AB_ produced a bit more dopamine than those derived from iPSCs that had been grown in E8 and both produced considerably more dopamine than the embryonic stem cells that we tested. However, the unexpected result was that the dopaminergic neurons derived from NME7_AB_ grown cells secreted significant amounts of dopamine at Day 30, 16.90 ng/mL, whereas the dopaminergic neurons derived from cells grown in E8 media at Day 30 only expressed 1.77 ng/mL. The early, Day 30 production of dopamine could be beneficial from a regulatory affairs standpoint, as it serves as a functional characterization of the cellular therapeutic at a timepoint considered to be optimal for implantation. Previous work showed that dopaminergic neuron precursors, implanted into the brain at about Day 28 engrafted better and had more of a therapeutic benefit than those implanted at Day 40–60 [32].

### The effect of stimulating versus inhibiting the β-catenin pathway on mesendoderm differentiation

Mesenchymal stem cells (MSCs) have been shown to have therapeutic benefit, particularly for the treatment of stroke and heart disease [33]. Most of the MSCs that are currently in clinical use are isolated from bone marrow [34–37], which is not a renewable source and introduces donor to donor variability. MSCs are finite cells [38], and senesce after a limited number of passages, depending on the source of the MSCs. Bone marrow derived MSCs senesce after about 65 days in culture and after 13–16 cumulative doublings [39,40]. Although one publication reported an embryonic stem cell line that didn’t senesce until after 60 days and 50 doublings [41], it is appreciated in the field that deriving MSCs from pluripotent stem cells is difficult and deriving MSCs from iPSCs is even more problematic. Reproducibility [42], clonal restriction [43], and premature senescence [38] are problems that have thus far plagued the utility of stem cell-derived MSCs.

Indicators of successful differentiation to MSCs are: 1) the ability to grow on bare plastic; 2) resistance to early senescence; and 3) the ability to differentiate into chondrocytes, adipocytes and osteogenic cells, also known as tri-lineage differentiation. Here, we differentiated pluripotent stem cells, grown in several different media, into MSCs using the protocol of Kimbrel et al. [40] (S2A Fig), which had been shown to work for an embryonic stem cell (ESC) line [41]. We compared MSC generation from iPSCs that had been grown in either NME7_AB_ naïve media, E8 or mTeSR1, plus or minus a β-catenin agonist or inhibitor. For comparison to the earlier work of Kimbrel et al., we also included MSCs derived from an ESC line that was grown in FGF2 media over a feeder layer of mouse embryonic fibroblasts (MEFs). Because two different iPS clones that had been cultured in E8 failed at the growth on Matrigel stage, we also included iPSCs that had been cultured in FGF2 over MEFs. iPSCs that had been grown in NME7_AB_ media, ESCs that had been grown in FGF2 medium over MEFs, and iPSCs grown in mTeSR1 plus WNT3A all successfully differentiated to MSCs with the characteristic morphology of each stage of development from the grape-like clusters of hemangioblasts, to the fibroblast-like morphology when grown on Matrigel and plastic (S2B Fig). Those MSCs had sustained ability to grow on bare plastic, but iPSCs previously grown in FGF2 media began to senesce with the initial growth on plastic (S2C Fig).

**Fig 6:**
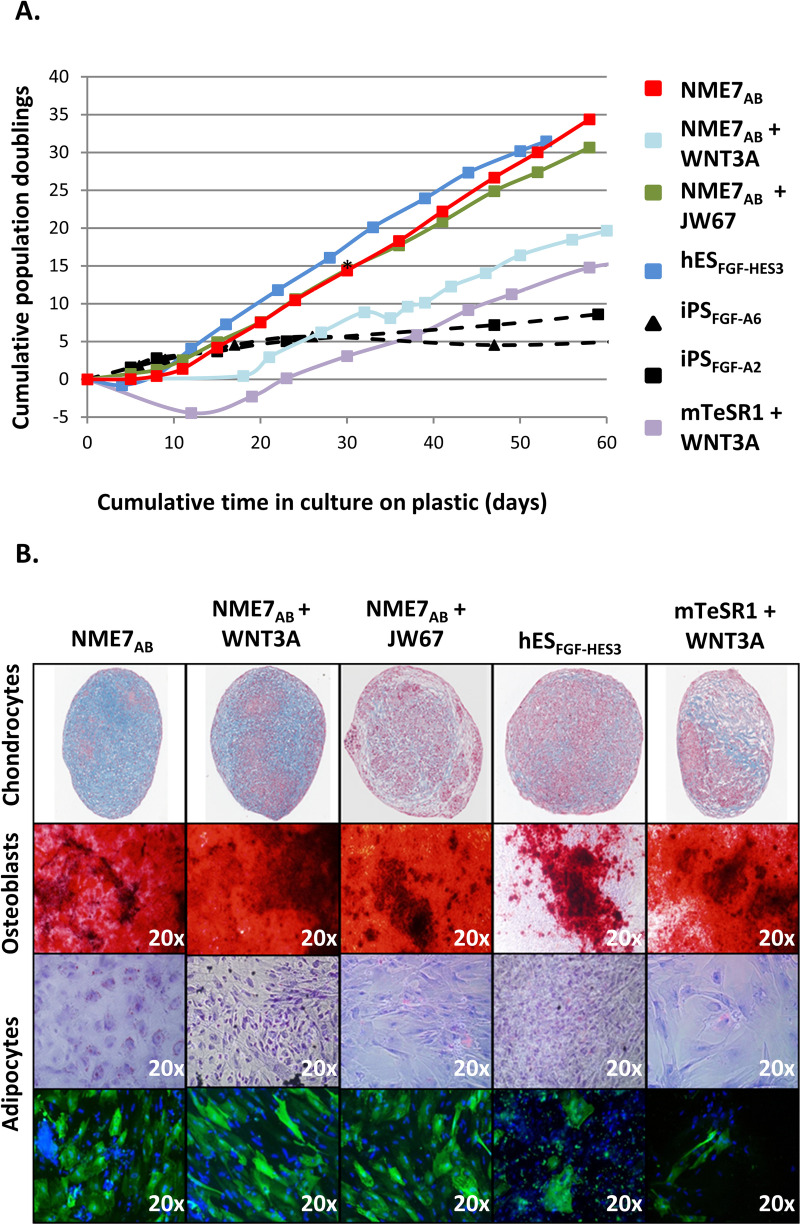
Stimulating versus inhibiting the **β-catenin signaling in mesenchymal stem cell differentiation.** The quality of mesenchymal stem cells (MSCs) derived from various types of pluripotent stem cells that had been cultured in different media, in the presence or absence of a β-catenin agonist or inhibitor was assessed and subsequent trilineage differentiation was superior when starting iPSCs were cultured in NME7_AB_ naïve media versus E8, mTeSR1 or in FGF2 over MEFs. In some conditions, β-catenin agonist WNT3A or inhibitor JW67 were added for 48 hours before initiating differentiation protocol. **(A)** The ability of the MSCs to proliferate without senescing was measured and plotted as cumulative doublings as a function of time in culture. **(B)** MSCs differentiated from pluripotent stem cells that had been cultured in various media as indicated were tested for their ability to differentiate into chondrocytes (Alcian Blue), osteoblasts (Alizarin Red) and adipocytes (Oil Red O and anti-FABP4).

The ability of the MSCs to resist senescence was tested by extended growth on plastic, where cumulative population doublings as a function of time were measured (Fig 6A). The embryonic stem cells grown in FGF2 over MEFs differentiated to MSCs that had 32 population doublings in 53 days. MSCs derived from iPSCs that had been grown in NME7_AB_ media had 35 doublings in 58 days. MSCs from iPSCs previously grown in mTeSR1 plus WNT3A doubled 15-times in 58 days and MSCs from iPSCs grown in FGF2 did not significantly multiply after the fifth population doubling.

The MSCs that were capable of sustained growth on plastic were then tested for their ability to differentiate down three lineages into chondrocytes, osteoblasts, and adipocytes (Fig 6B) [40]. Successful differentiation to chondrocytes was assessed by staining with Alcian Blue that detects the matrix deposition of sulfated glycosaminoglycans. Differentiation to osteoblasts was determined by staining with Alizarin Red that detects calcified extracellular matrix. Adipocyte cultures were stained with Oil Red O to detect lipid accumulation and also stained with an antibody to detect FABP4, a fatty acid binding protein found in adipocytes, and Hoechst to stain the nuclei. The MSCs derived from ES cells grown in FGF2/MEFs or mTeSR1/WNT3A did not convincingly form adipocytes as they lack clear accumulation of lipid droplets and the staining for FABP4 is not often associated with a cell. MSCs derived from iPSCs that had been grown in NME7_AB_ media without β-catenin agonist or inhibitor appear to form the best chondrocytes, osteoblasts, and adipocytes. NME7_AB_ grown iPSCs, with or without the addition of a β-catenin agonist or inhibitor consistently differentiated into MSCs that expanded well, resisted senescing and showed superior tri-lineage differentiation.

We next tested two protocols for generating hepatocytes from iPSCs. Hepatocytes were differentiated from human iPSCs that had previously been grown in NME7_AB_, E8 or mTeSR1 according to the Kajiwara protocol [44] or the Cai protocol [45] (S3 Fig). The Kajiwara protocol was of interest to us because it begins with seven days of activating the Wnt/β-catenin pathway with exogenous WNT3A.

**Fig 7:**
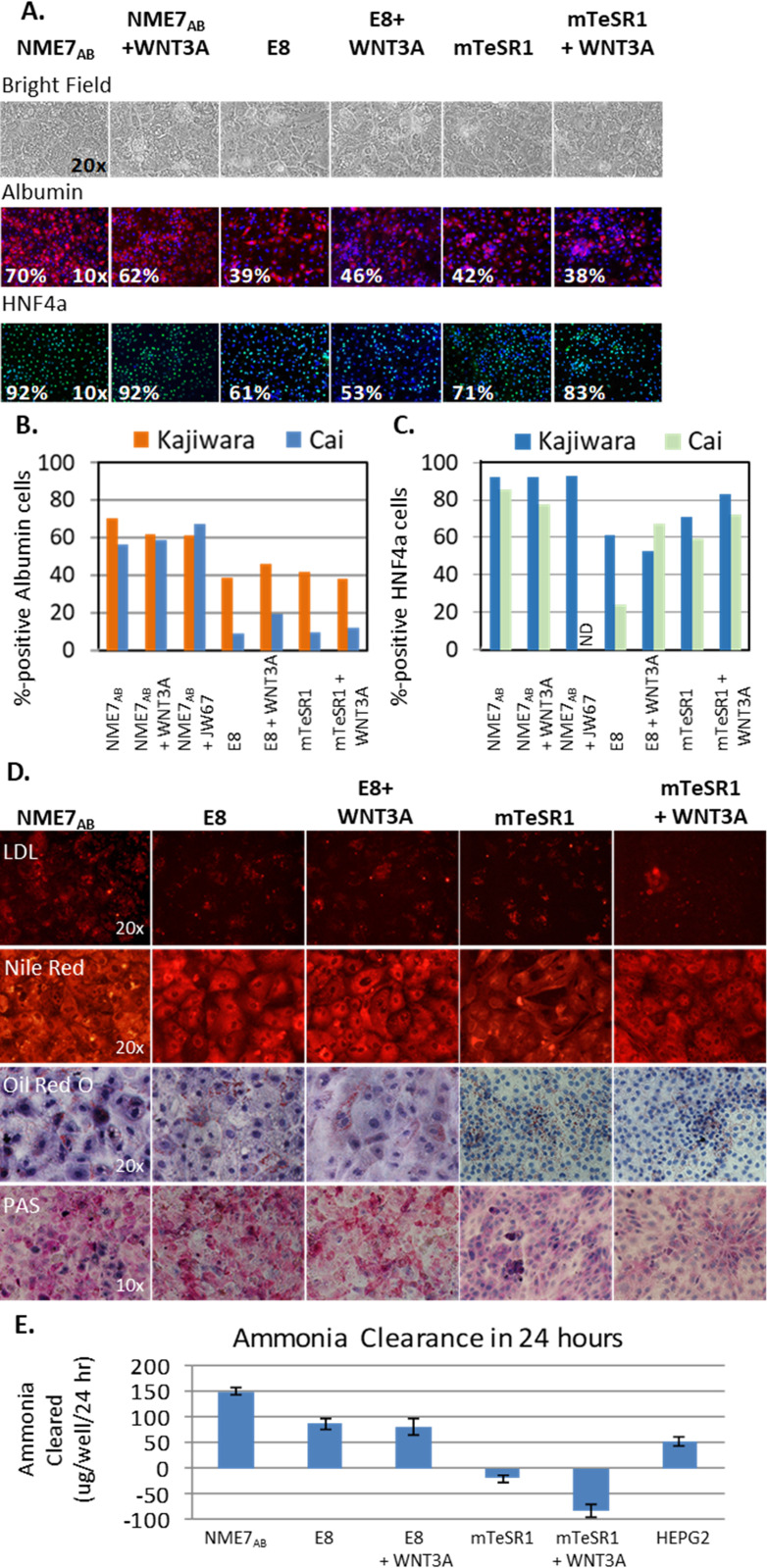
Stimulating versus inhibiting **β-catenin signaling in hepatocyte differentiation.** iPSCs that had been cultured in either NME7_AB_ naïve media, E8 or mTeSR1, in the presence or absence of WNT3A, a β-catenin agonist, were differentiated into hepatocytes. Two different protocols were followed: Kajiwara that begins with the addition of WNT3A over several days or the Cai protocol. **(A)** Phase contrast images taken at Day 21 of the Kajiwara protocol show characteristic hepatic morphology as well as evidence of lipid droplets and binucleated cells. Cells expressing albumin (red) or HNF4a (Green) were detected by immunofluorescence. **(B)** A graph of quantification of immunofluorescent images, compares the percent albumin positive cells produced by the Kajiwara protocol versus the Cai protocol at Day 20. **(C)** A graph of quantification of immunofluorescent images, compares the percent HNF4a positive cells produced by the Kajiwara protocol versus the Cai protocol at Day 20. **(D)** Functional assays were performed on hepatocytes generated using the Kajiwara protocol. The ability of the cells to take up low‐density lipoprotein was detected using a fluoresceinated LDL. Their ability to store lipids was assessed using a Nile Red and Oil Red O staining. The ability of the resultant cells to store glycogen by Periodic Acid‐Schiff (PAS) staining was also assessed. **(E)** Ammonia degradation (conversion in glutamine synthesis) is a function of parenchymal hepatocytes. The results of an ammonia clearance assay are shown.

When using the Kajiwara protocol, cells that had been previously grown in NME7_AB_, E8 or mTeSR1 all consistently differentiated into albumin positive and HNF4a positive cells that had the geometric shape, as well as evidence of lipid droplets and binucleated cells, that are characteristic of hepatocytes (Fig 7A). Cells expressing albumin or HNF4a were detected by immunofluorescence and the percentage of cells expressing that marker was quantified. In contrast, using the Cai protocol, iPSCs that had been grown in E8 or mTeSR1 failed to differentiate into albumin-producing hepatocyte progenitors more than 50% of the time. The addition of WNT3A to E8 and mTeSR1 media for 48 hours before initiating the Cai protocol, decreased the frequency of failures, but the percentage of resultant cells that produced albumin was far less than that of cells differentiated using the Kajiwara protocol (Fig 7B). The addition of WNT3A to iPSCs previously grown in E8 did rescue expression of HNF4a (Fig 7C). Neither the addition of β-catenin agonist WNT3A, nor inhibitor JW67, had a significant effect on hepatocytes derived from cells that had been cultured in NME7_AB_ media.

Functional assays were performed on the hepatic cells produced by the Kajiwara protocol. Their capacity to take up LDL [46], to store lipids by both Nile Red [47] and Oil Red O [46] assays, and to store glycogen by periodic acid-Schiff assay [44] was assessed (Fig 7D). Hepatocytes derived from all the starting stem cell conditions appear to adequately take up low‐density lipoprotein and store lipids as assessed by Nile Red staining. However, cells that had been grown in mTeSR1, with or without WNT3A, do not show evidence of lipid storage in an Oil Red O staining assay and similarly performed poorly in the Periodic Acid‐Schiff (PAS) assay to assess glycogen storage. Because the ability to rid the body of toxins is an important function of hepatocytes, ammonia clearance was also tested (Fig 7E) [44]. Resultant hepatocytes appeared to be comparable, except that cells that had previously been grown in mTeSR1 were deficient in their ability to clear ammonia, compared to those derived from stem cells that had been grown in NME7_AB_ or E8.

## Discussion

The finding reported here that NME7_AB_ grown naïve stem cells have the highest level of active β-catenin raised the interesting question of whether or not activation of the Wnt/β-catenin pathway alone could induce a naïve or naïve-like stem cell state. This idea gained traction when we reviewed two published protocols, where differentiation to mesendoderm and to neuroectoderm had been improved by beginning with activation of the Wnt/β-catenin pathway. Kajiwara et al. [44] differentiated primed state stem cells to hepatocytes using a protocol that begins with the addition of WNT3A over several days. Kim et al. [30] improved differentiation to dopaminergic neurons using a protocol that starts with the addition of CHIR99021, an indirect agonist of the Wnt/β-catenin pathway, over several days. These two examples conflict with the report [14] that pluripotent stem cells expressing low levels of active β-catenin differentiate down neuroectoderm lineage better than cells expressing high levels of β-catenin. Indeed, here we showed that in the absence of other growth factors, added WNT3A gave rise to islands of apparently naïve cells in a sea of primed state or differentiating cells. The islands consisted of cells that were OCT4 positive and had two active X chromosomes, as evidenced by their lack of the punctate staining of H3K27 methylation, which would have indicated inactivation of one X chromosome. Interestingly, a figure from Kajiwara et al. [44] also shows two segregated populations of OCT4^+^ and OCT4^-^ cells when they add WNT3A before initiating differentiation. They found that the iPSC clone that exhibited the segregated populations differentiated to the highest albumin secreting hepatocytes, whereas a “sibling” clone that had fewer OCT4 + cells after WNT3A addition formed hepatocytes with poor albumin secretion.

We note that in these experiments, activating β-catenin by pre-treatment with agonist WNT3A improved the differentiation of primed state stem cells to neuroectoderm and mesendoderm, but had essentially no effect on NME7_AB_ naïve stem cells. This result is consistent with the idea that WNT3A improved differentiation of primed state cells by inducing a sub-population to revert to a naïve-like state [16,48]. The other possibility that differentiation was improved by the OCT4-negative population being poised to differentiate, seems unlikely since the homogeneous population of NME7_AB_ naïve cells consistently out-performed the primed state cells with or without pre-treatment with WNT3A or CHIR99021.

It is intriguing, though, that stimulation of the Wnt/β-catenin pathway did not result in a homogeneous population of naïve-like stem cells nor a uniformly mixed population of differentiating and pluripotent stem cells. Instead, the activation of the Wnt/β-catenin pathway by the addition of WNT3A appears to, by an unknown mechanism, trigger a randomized decision to either self-replicate or differentiate. In some cases, levels of active β-catenin were increased by the addition of a small molecule inhibitor, raising the possibility of action against secondary targets, which could have produced multiple downstream effects. The molecular basis for the same condition causing one population of cells to differentiate while causing another population to apparently revert to an earlier naïve-like state is a topic for future research. The purpose of having two segregated populations is also unclear; perhaps the naïve-like population represents a reserve pool of self-replicating cells to draw from for future differentiation to other cell types.

In future work, it would be interesting to separate these two populations and determine their differentiation potential in the absence of the other. It would also be informative to label the exogenously added WNT3A and by single cell analysis determine if there is a first level of β-catenin that induces expression of a first set of genes and second level of β-catenin that induces expression of a second set of genes.

## Supporting information

S1 Table List of antibodies and assay kits used in the experiments. (DOCX)

S1 Fig (A) Barnea-Cramer protocol schematic used to generate iPS-derived retinal progenitor cells. (B) Overlay of fluorescent images stained for early marker RX1 and CHX10 that specifies developmental fates toward the retinal progenitor cells and the retina in particular. Photographs taken on Day 30 of differentiation. (C) Flow cytometry scatter plots measuring the percent PAX6 positive cells for each condition at Day 13 show delayed expression of PAX6 in cells that had been cultured in E8, which is corrected by the addition of β-catenin agonist WNT3A.
(TIF)

S2 Fig (A) Protocol used to generate iPS-derived mesenchymal stem cells. (B) Phase contrast images show the morphology of stem cells differentiated into MSCs in chronological order of their progression from pluripotency to embryoid bodies, to hemangioblasts to growth on Matrigel and finally to growth on bare plastic at passage 5. Multiple attempts to differentiate mTeSR1 and E8 grown iPSCs into MSCs failed at the stage of growth on Matrigel. (C) Number of days before cells were confluent at passage numbers 4 and 5 for different media conditions.
(TIF)

S3 Fig (A) Cartoon of the Kajiwara protocol for generating iPS-derived hepatocytes, which starts with the addition of β-catenin agonist WNT3A for the first 8 days. (B) Cartoon of the Cai protocol used to generate iPS-derived hepatocytes.
(TIF)

S1 File This file contains the original raw data in Excel format. (XLSX)

S2 File Original WB blots. (PPTX)
