# Supplementary material for: The Wnt pathway induces a naïve-like subpopulation in primed stem cells, while NME7AB leads to a homogeneous naïve-like population
Source: PLoS One. 2025 Jun 25;20(6):e0325997. doi: 10.1371/journal.pone.0325997 (PMC12193845; doi:10.1371/journal.pone.0325997)
Supplement: S1 Table — (DOCX) [file pone.0325997.s001.docx]

| **Antibody** | **Cat No** | **Vendor** |
| --- | --- | --- |
| OCT4 | SC-5279 | Santa Cruz |
| H3K27me | C36B11 | Cell Signaling |
| PAX6 | MA1-109 | Thermo Fisher |
| RX1 | Ab23340 | Abcam |
| CHX10 | Ab16141 | Abcam |
| MITF | PA538294 | Thermo Fisher |
| FABP4 | AF3150-SP | R&D Systems |
| Albumin | CL2513A | Cedarlane |
| HNF4a | SC-6556 | Santa Cruz |
| LDL | L3482 | Thermo Fisher |
| GIRK2 | ab65096 | Abcam |
| TH | ab112 | Abcam |
| DAT | PA1-4656 | Thermo Fisher |
| Tuj1 | MA1-118 | Thermo Fisher |
|  |  |  |
| **Assays** |  |  |
| Nile Red | ab228553 | Abcam |
| Ammonia Assay Kit | 295-78901 | Wako |
| Periodic Acid-Schiff Kit | 395b | Sigma-Aldrich |
| Alcian Blue | NA | NA |
| Alizarin Red | TMS-008-C | Sigma-Aldrich |
| Oil Red O | MAK194 | Sigma-Aldrich |
