# Supplementary figures and images for: The Wnt pathway induces a naïve-like subpopulation in primed stem cells, while NME7AB leads to a homogeneous naïve-like population
Source: PLoS One. 2025 Jun 25;20(6):e0325997. doi: 10.1371/journal.pone.0325997 (PMC12193845; doi:10.1371/journal.pone.0325997)

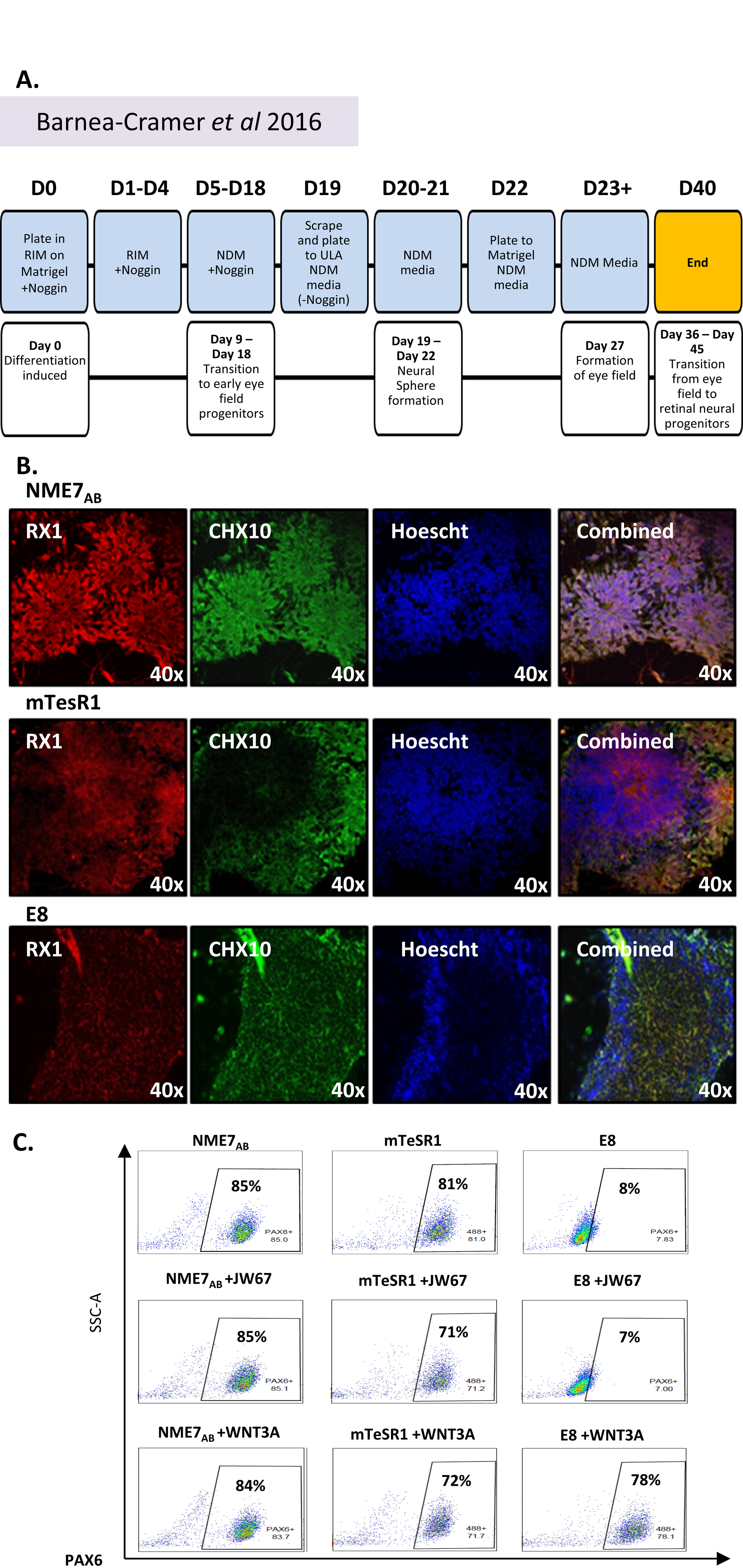

Supplement: S1 Fig — (B) Overlay of fluorescent images stained for early marker RX1 and CHX10 that specifies developmental fates toward the retinal progenitor cells and the retina in particular. Photographs taken on Day 30 of differentiation. (C) Flow cytometry scatter plots measuring the percent PAX6 positive cells for each condition at Day 13 show delayed expression of PAX6 in cells that had been cultured in E8, which is corrected by the addition of β-catenin agonist WNT3A. (TIF) [file pone.0325997.s002.tif]

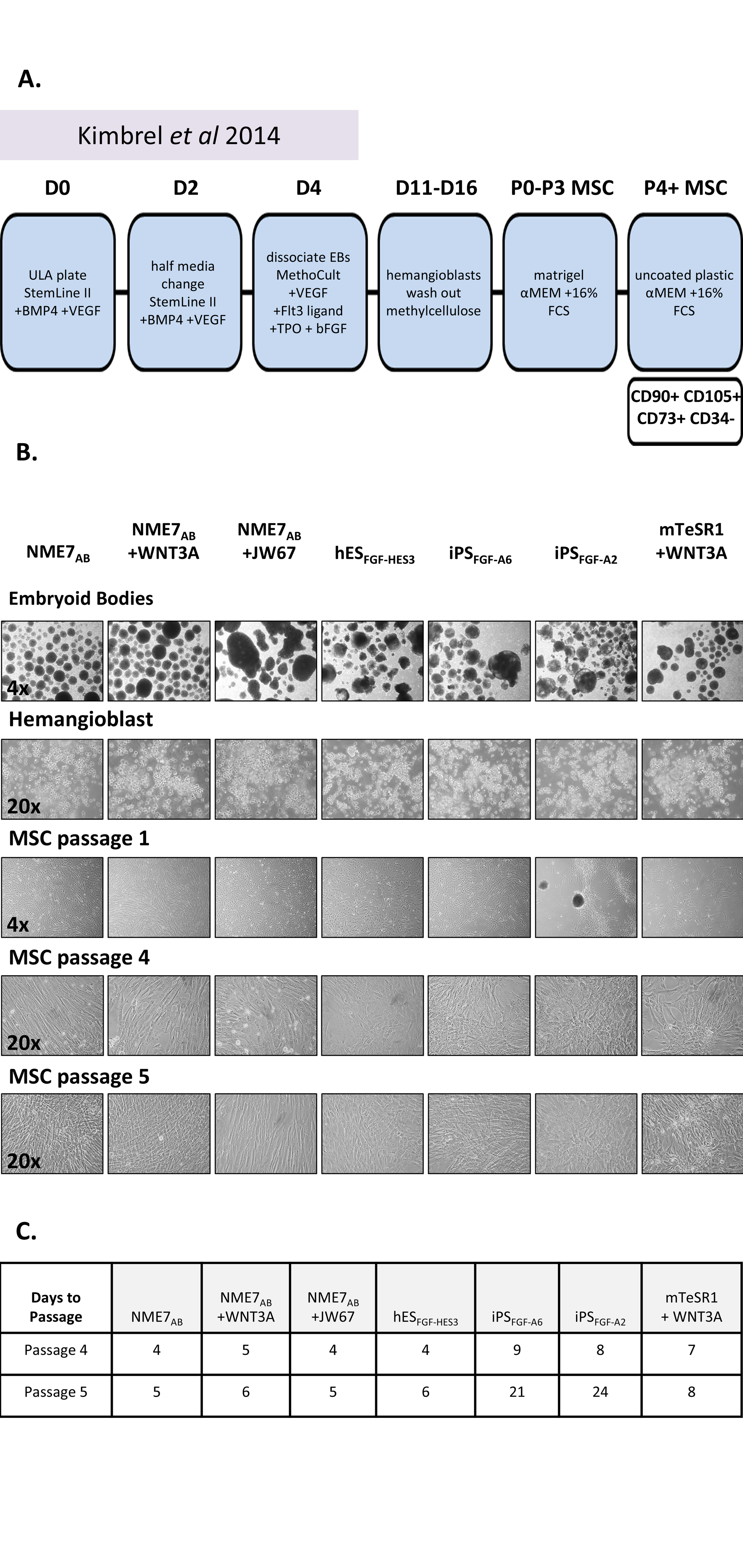

Supplement: S2 Fig — (B) Phase contrast images show the morphology of stem cells differentiated into MSCs in chronological order of their progression from pluripotency to embryoid bodies, to hemangioblasts to growth on Matrigel and finally to growth on bare plastic at passage 5. Multiple attempts to differentiate mTeSR1 and E8 grown iPSCs into MSCs failed at the stage of growth on Matrigel. (C) Number of days before cells were confluent at passage numbers 4 and 5 for different media conditions. (TIF) [file pone.0325997.s003.tif]

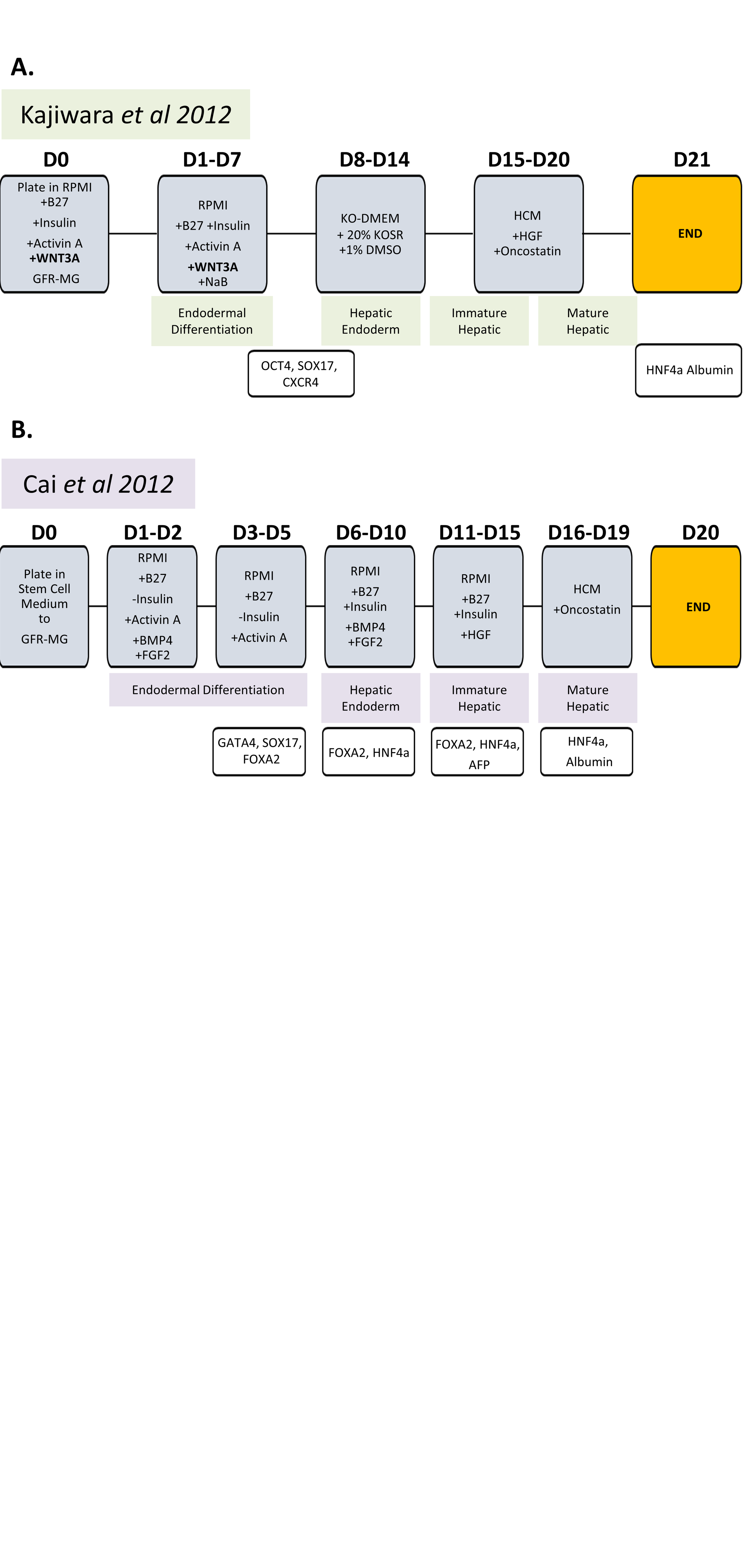

Supplement: S3 Fig — (B) Cartoon of the Cai protocol used to generate iPS-derived hepatocytes. (TIF) [file pone.0325997.s004.tif]
