# Supplementary material for: The Wnt pathway induces a naïve-like subpopulation in primed stem cells, while NME7AB leads to a homogeneous naïve-like population
Source: PLoS One. 2025 Jun 25;20(6):e0325997. doi: 10.1371/journal.pone.0325997 (PMC12193845; doi:10.1371/journal.pone.0325997)
Supplement: S2 File — (PPTX) [file pone.0325997.s006.pptx]

## Slide 1
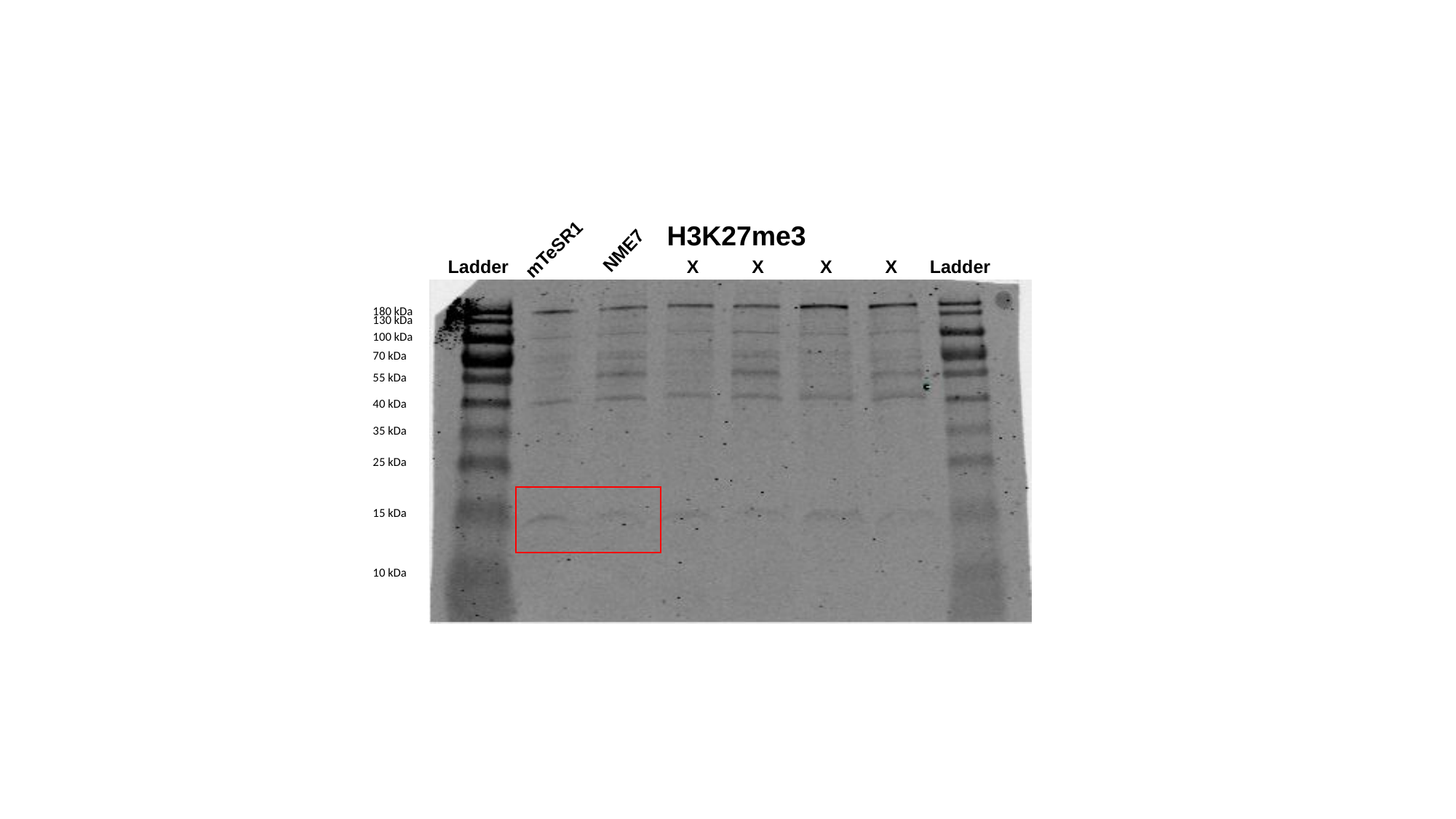

mTeSR1
NME7
Ladder
X
X
X
X
Ladder
H3K27me3
180 kDa
130 kDa
100 kDa
70 kDa
55 kDa
40 kDa
35 kDa
25 kDa
15 kDa
10 kDa

## Slide 2
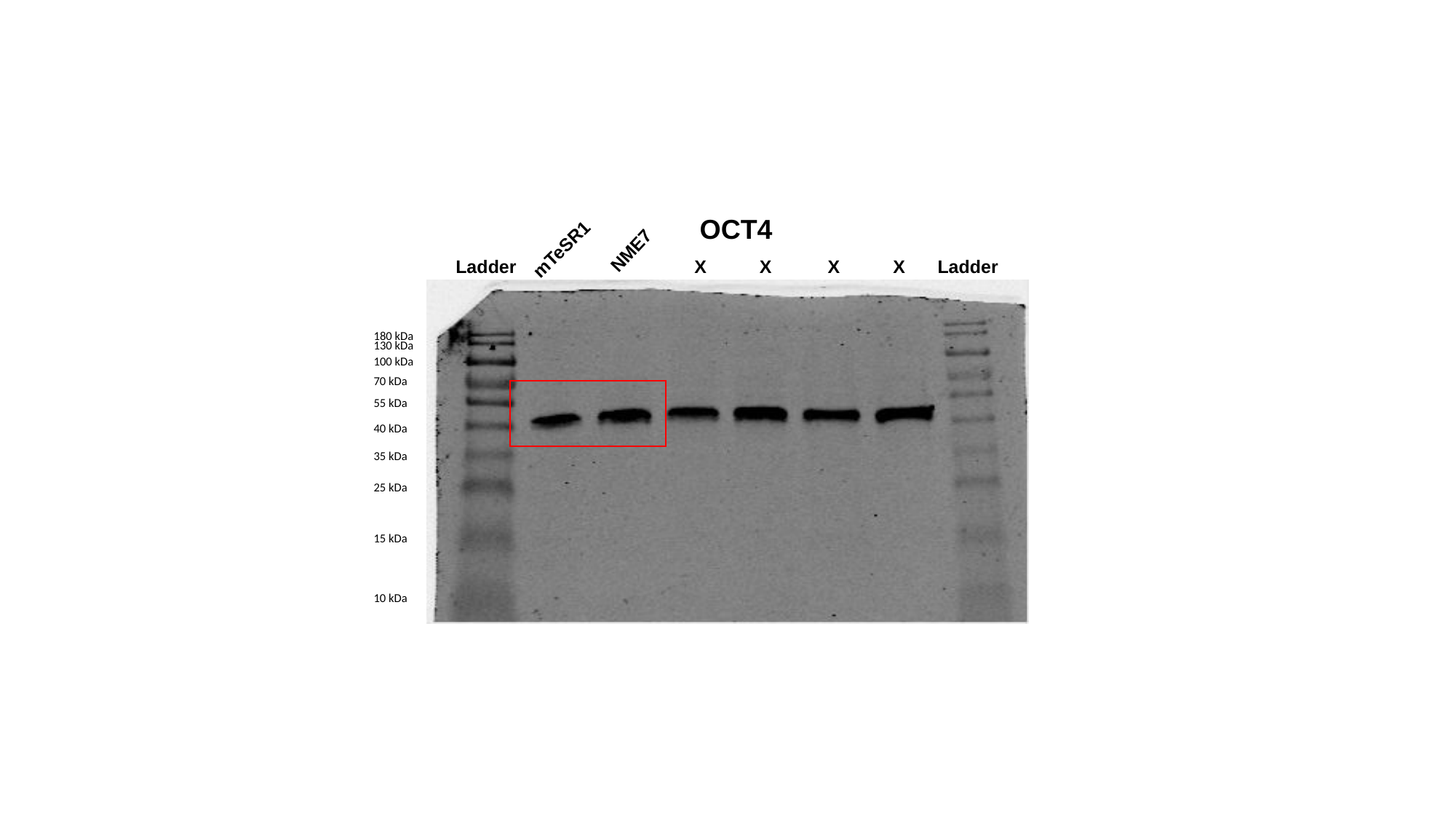

mTeSR1
NME7
Ladder
X
X
X
X
Ladder
OCT4
180 kDa
130 kDa
100 kDa
70 kDa
55 kDa
40 kDa
35 kDa
25 kDa
15 kDa
10 kDa

## Slide 3
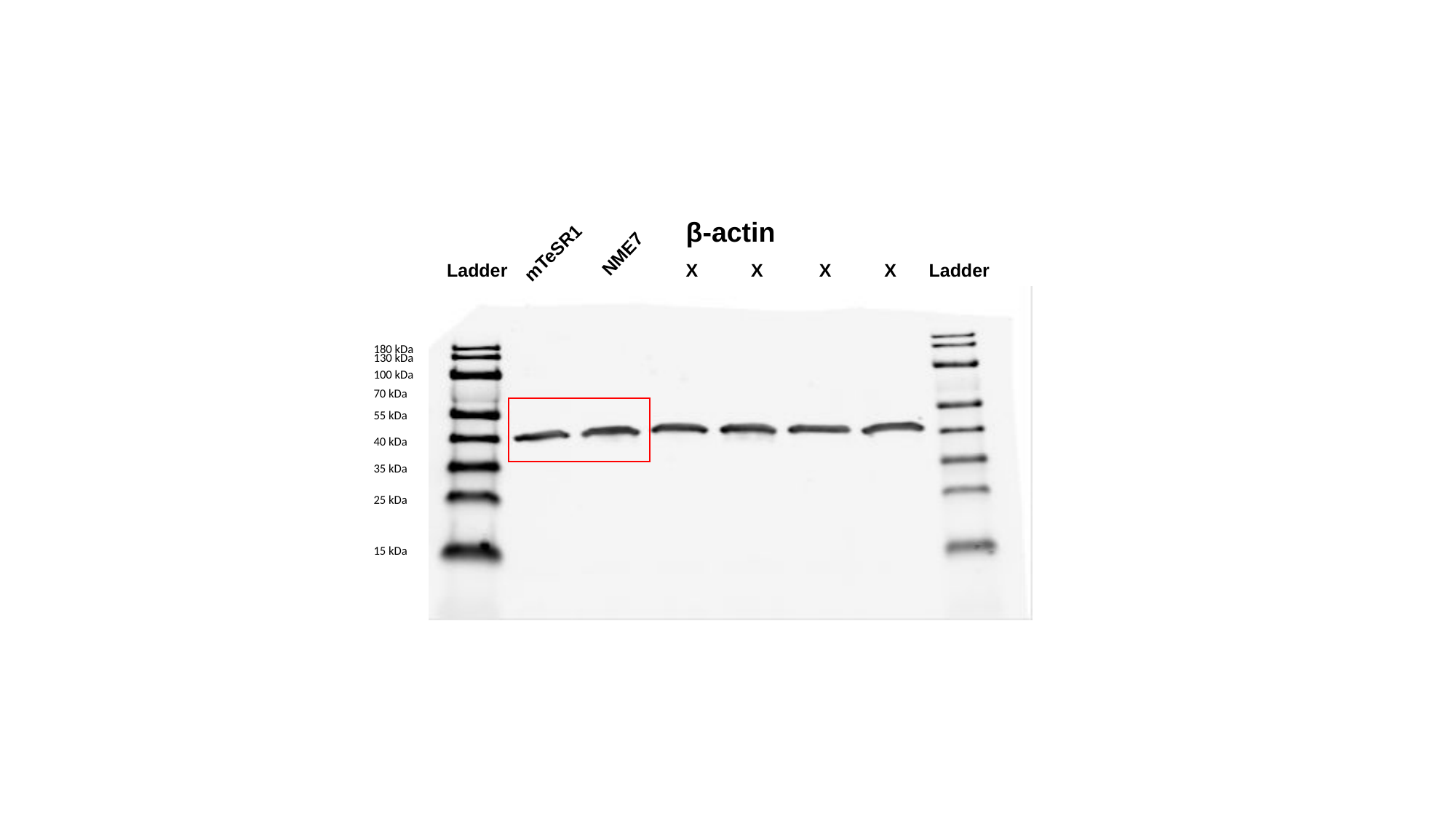

mTeSR1
NME7
Ladder
X
X
X
X
Ladder
β-actin
180 kDa
130 kDa
100 kDa
70 kDa
55 kDa
40 kDa
35 kDa
25 kDa
15 kDa
